# The distribution of bushmeat mammals in unflooded forests of the Central Amazon is influenced by poaching proxies

**DOI:** 10.1002/ece3.10783

**Published:** 2023-12-03

**Authors:** Gilson de Souza Ferreira Neto, Fabricio Beggiato Baccaro, Matthew J. Phillips, Rodrigo Lima Massara

**Affiliations:** ^1^ Programa de Pós‐Graduação em Ecologia/INPA‐V8 INPA—Instituto Nacional de Pesquisas da Amazônia Manaus Brazil; ^2^ Departamento de Biologia, Instituto de Ciências Biológicas Universidade Federal do Amazonas Manaus Brazil; ^3^ School of Earth, Environmental and Biological Sciences Queensland University of Technology Brisbane Queensland Australia; ^4^ Laboratório de Ecologia e Conservação, Departamento de Genética, Ecologia e Evolução, Instituto de Ciências Biológicas Universidade Federal de Minas Gerais Belo Horizonte Brazil

**Keywords:** body mass, distance to settlements, game species, number of residents, occupancy modelling, rainforest mammals

## Abstract

Medium to large rainforest mammals are key conservation flagship groups that offer non‐redundant ecosystem functions, but anthropic pressures, such as illegal hunting, may strongly affect their occupancy in Amazonia. We combined camera traps and occupancy models to assess the influence of distance from human settlements, the number of families per settlement and the synergetic effect of the average weight of 27 species on the occupancy probability of mammals. Specifically, we classified mammal species according to the game preferences of hunters (i.e. a group of species depleted for bushmeat, a group of species hunted for retaliation and a group of non‐hunted species). We also accounted for the influence on the detection probability of each group of both the number of days each camera operated and the body weight of mammals. The occupancy probability of the bushmeat group (i.e. deer, peccaries, agoutis, pacas and armadillos) was lower at locations closer to human settlements. Still, the number of families correlated positively with occupancy, with the occupancy probability of the group being slightly higher at sites with more families. This difference was probably due to larger and more abundant crops and fruiting trees attracting wildlife at such sites. Conversely, the occupancy probability of the retaliation group (i.e. carnivores) and the non‐hunted group (i.e. opossums, spiny rats, squirrels and anteaters) were indifferent to anthropogenic stressors. The detection probability of the non‐hunted and particularly the most depleted species correlated negatively with body weight. This may suggest that larger species, especially those from the bushmeat group, are rarer or less abundant in the system, possibly because they are the preferable target of hunters. In the long term, locals will likely need to travel long distances to find harvest meat. Poaching also threatens food security since game bushmeat is an essential source of protein for isolated rural Amazonians.

## Graphical abstract

Our study focuses on investigating the impact of anthropogenic factors on the occupancy of medium and large mammals in unflooded forests of the Central Amazon. Through a camera trapping survey conducted across 47 sampling sites during the dry season, we provide a comprehensive analysis of the influence of anthropogenic stressors on mammal occupancy patterns in terra‐firme forests. Our findings reveal compelling evidence that anthropogenic stressors have a strong influence on mammal occupancy, particularly with regard to game preference in the study area.

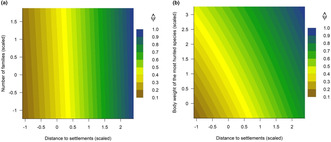

## 1 INTRODUCTION

The Neotropic realm is the global cornerstone of mammal diversity, accounting for one‐quarter of all species worldwide (Burgin et al., 2018). For instance, Brazil harbours 751 native species (≈ 223 endemics), representing ~12% of all mammal species currently described (Quintela et al., 2020). In Brazil, non‐volant mammals of the Amazon represent ~70% of all medium to large species (Reis et al., 2008). Still, many of these species are threatened by anthropogenic disturbances, including the replacement of the forest for huge‐scale agribusiness activities, timber and poaching (Pacheco et al., 2021). In many cases, hunting is a non‐legal action, not sustainable and, in some cases, not even necessary for local subsistence (Machado et al., 2008; Quintero et al., 2023).

Various anthropogenic stressors affect wildlife and may be deleterious to some species (Benítez‐Lopez et al., 2017). Proxies for anthropogenic impacts such as distance from communities (Levi et al., 2011) and the associated number of inhabitants (Gonedelé‐Bi et al., 2022; Laurance et al., 2002) are frequently used as an indicator for poaching pressure and therefore could indicate wildlife status (Roopsind et al., 2017). Overall, hunters prefer to hunt within a 20‐km radius of human settlements (Benítez‐Lopez et al., 2017), so the optimal foraging for human settlements means more units of prey possible with less effort and fewer resources employed (Benítez‐Lopez et al., 2017). That implies that the hunting intensity leads to resource depletion of target species in sites closer to communities, especially in the nearest 5 km to settlements (Pérez‐Flores et al., 2022). In addition to clandestine hunting, the presence of human communities and local population density may also affect mammal occupancy through other indirect anthropogenic effects, such as increases in wildfires (Barlow & Peres, 2006), deforestation (Laurance et al., 2002), timber production, slash‐and‐burn monoculture and livestock (Beirne et al., 2019), which therefore, may also decrease fruit production and habitat use for native wildlife (Barlow & Peres, 2006).

Given that poaching is not random and is one of the main disturbances of anthropic disturbances, with some species being preferentially hunted (Peres, 2000), sites closer to human settlements may experience a more abrupt change in mammal community structure (Mesquita & Barreto, 2015; Silveira et al., 2008). For example, in the Amazon basin, distance to settlements had a negative but weak influence on the occupancy probability of ocelots (Wang et al., 2019). It is known that carnivorous species might be hunted for retaliation due to human–carnivore conflict in order to avoid economic losses as they feed on domestic livestock and for the supposed safety of residents (Cavalcanti et al., 2010; Jędrzejewski et al., 2017). For instance, some predatory species, such as pumas and ocelots, have been negatively associated with livestock species (e.g. chickens), which increases the overlap and conflict with humans, as reported in the Eastern Brazilian Amazon (Whiteman et al., 2007). Furthermore, in the Ecuadorian Amazon, the further the distance from settlements, the higher the occurrence of jaguar prey such as ungulates, which indicates the depletion of prey availability for predators close to human settlements (Espinosa et al., 2018), likely increasing human–carnivore conflict.

In addition to hunting preferences for some species, body mass has a synergetic effect because hunters often prefer larger species to obtain a greater energetic return (Peres et al., 2016). The distribution of mammals around human settlements may indicate the species' resilience to anthropogenic disturbances (Adhikari et al., 2019). Hunting is not random, with some species being preferentially hunted for bushmeat, mainly medium to large mammals (>20 kg), which have higher levels of defaunation than other mammal groups (Benítez‐López et al., 2019). On a global scale, 60% of the larger mammals are at risk due to hunting for human consumption (Ripple et al., 2016). In the Amazon, the occurrence of larger mammals is usually associated with low anthropogenic impacts (Peres et al., 2016), since they are usually the most depleted species (Scabin & Peres, 2021). The reduction or extirpation of larger mammals allows smaller and potentially competing species to exploit the surplus resources and increase their densities locally (Gutiérrez‐Granados & Dirzo, 2021). This pattern was first predicted by the compensation hypothesis in island faunas (Crowell, 1962; MacArthur et al., 1972) due to lower competition (Gil‐Sánchez et al., 2021) or mesopredator release (Jachowski et al., 2020). Still, mainland studies also underscore that anthropogenic stressors can lead to the extirpation of larger species and their replacement by smaller species (Peres & Dolman, 2000). Studies have shown the relationship between distance to settlements and the decay in large mammal distribution in different parts of the Amazon basin and worldwide. Some remarkable examples in the Amazon basin are in the Northern Brazilian Amazon (Melo et al., 2015), Eastern Brazilian Amazon (Mesquita & Barreto, 2015) or Peruvian Amazonia (Ohl‐Schacherer et al., 2007), as well as other parts of the world, including global biodiversity hotspots such as East Africa (Cavada et al., 2019), Thailand (Ngoprasert et al., 2007), Sumatra (Widodo et al., 2022), Malaysian Borneo (Deith & Brodie, 2020) and Western Nepal (Adhikari et al., 2019). Some studies have even reported that distance to human settlements is a robust predictor of poaching that is more important than forest management in regions such as Central Africa (Lhoest et al., 2020), in the sense that areas further away from settlements, and therefore less degraded, would act as a source of individuals to repopulate more defaunated areas closer to settlements (Begazo & Bodmer, 1998). Despite the wealth of literature detailing species level occupancy probability, the synergetic effects of proxies of anthropogenic impacts interacting with mammal species body mass with different hunted groups remain in their infancy (Fernandes‐Ferreira & Alves, 2017), especially in some regions such as the Neotropical region and Southeast Asia (Ripple et al., 2016). Then, quantifying the importance of distance to settlements for mammals with different game preferences is crucial for shaping conservation approaches and identifying game species more vulnerable to poaching (Lhoest et al., 2020).

Here, we sampled terrestrial mammals across a large unflooded area (*terra‐firme* forests) in central Amazonia to evaluate the influence of anthropogenic stressors (i.e. distance from human settlement and number of families per settlement) on the occupancy probability of mammals subject to different poaching preferences. Additionally, we evaluated whether this relationship depends on species‐specific body mass. We expected occupancy probability to be influenced by distance to human settlements and the number of families, but species body weight also affects this relationship. Specifically, we predicted that the occupancy probability of most hunted and larger species would be higher at locations more distant from human settlements and with fewer inhabitants since these species are usually targeted for poaching. On the other hand, we also predicted that less depleted and smaller species would be more likely to occur at locations closer to human settlements and with more people due to the absence of the larger and most hunted species. Finally, we expected a negative relationship between detectability and species body weight, as larger species are either naturally rare in the system (e.g. apex predators) or less abundant due to being more highly preferred for poaching.

## 2 MATERIALS AND METHODS

### 2.1 Study area

**FIGURE 1:**
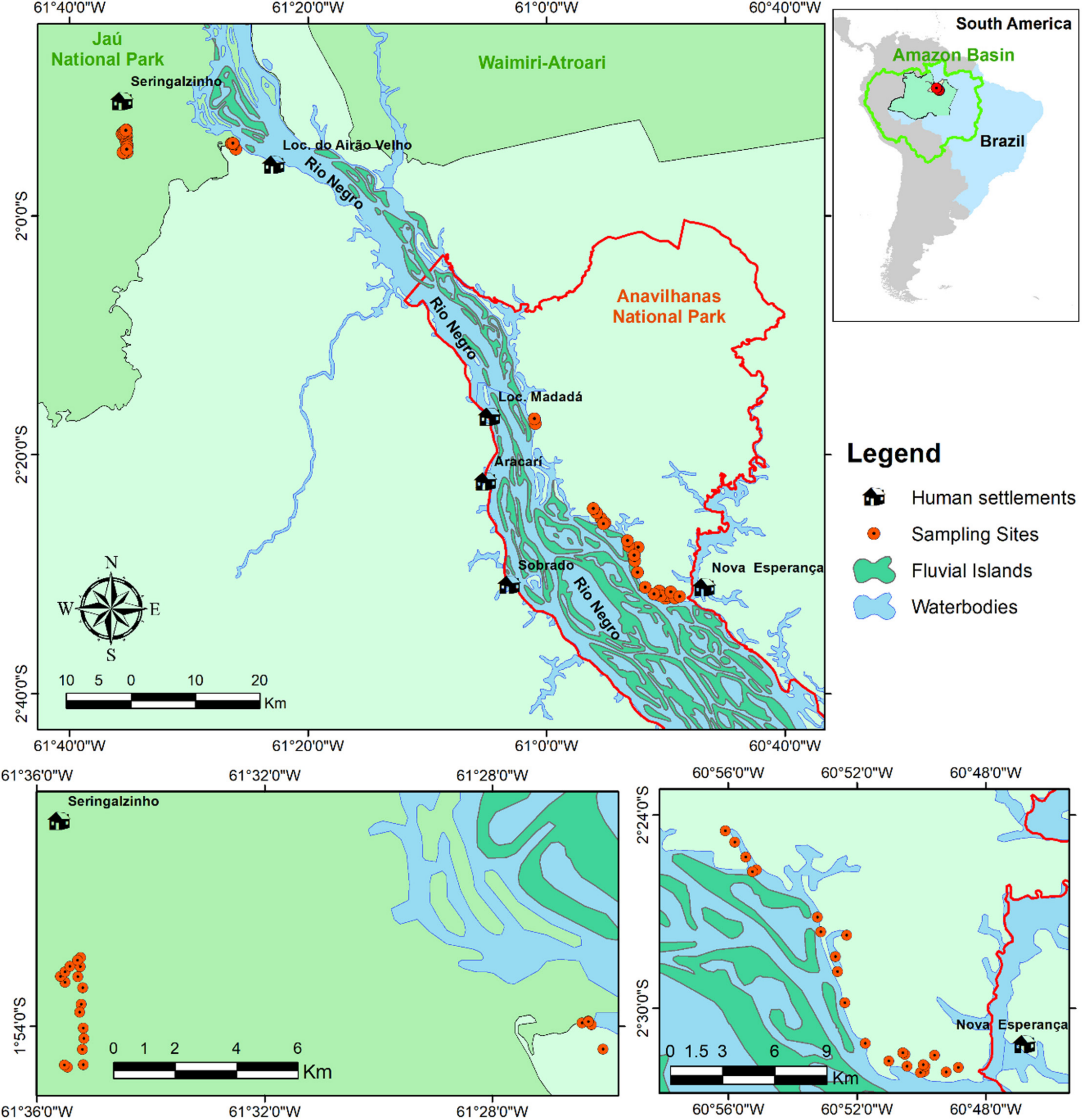
Map of the study area in the state of Amazonas, indicating the location of the 47 *terra‐firme* camera trap sites in the Anavilhanas and Jaú National parks in the lower Rio Negro Basin for sampling terrestrial mammals.

The study was carried out within two protected areas and surrounding sites on both margins of the Negro River in central Brazilian Amazon—Anavilhanas National Park and Jaú National Park (Figure 1). The Anavilhanas National Park covers an area of 350,470 hectares, located between the municipalities of Manaus and Novo Airão (ICMBio, 2017). Jaú National Park has an area of 2367.333 hectares, located between the municipalities of Novo Airão and Barcelos (ICMBio, 2017).

The human settlements occur in the areas of influence of the Jaú and Anavilhanas Parks. Around Anavilhanas, Jaú and adjacent areas, 46 permanent human settlements are established. Data with population density from each community was given accordingly with the most recent data from SEMA (Secretary of Amazonas State for the Environment). Most of the permanent inhabitants of the settlements identify themselves as local campesinos, caboclos or Indigenous people. In most communities, there are more males than females, and hunting is done mainly by males (Campos, 2008). The human settlements are inserted in different protected areas around and inside the Anavilhanas National Park (PNA) and Jaú National Park (PNJ), where hunting is not allowed.

### 2.2 Camera trap survey

From 8 August to 25 October 2022, 49 camera traps were deployed. We calculated the sampling effort by multiplying the number of camera traps by the total number of days each camera trap remained active. Because two camera traps failed to work during surveys, 47 sampling stations remained operative throughout the sampling period, resulting in 3666 full‐day trappings. The 47 camera traps were deployed in *terra‐firme* forests in Anavilhanas (25 camera traps) and Jaú National Park (22 camera traps). Camera traps in the Jaú National Park were located around four research trails (i.e. Trilha da Biodiversidade, Trilha do Pesquisador, Trilha do Itaubal and Trilha da Sumaúma). In Anavilhanas, camera traps were placed around *Trilha do Apuaú* and other sites. We used two camera trap models with similar features and spaced them arbitrarily, but at different distances from human settlements, either in Jaú and Anavilhanas National Parks.

Each camera was deployed at a height of 30–40 cm at each sampling station. All camera traps were programmed with a 30‐s time interval between photos. We fixed the camera trap to a tree without bait to avoid potential bias in the species detection rates (Rocha et al., 2016). They operated continually for 24 h/day from 69 to 78 days with no delay between subsequent triggers, which were standardised for all sites.

### 2.3 Poaching preference and body mass

We categorised species accordingly to the poaching preferences of the local communities based on previous poaching inventory studies (Campos, 2008; Pezzuti et al., 2004) and management plans of the surrounding protected areas (*RDS Rio Negro*, *RDS Puranga Conquista*, *Plano de Manejo Parna Anavilhanas*, *Parque Estadual Rio Negro Setor Sul* and *Parque Estadual do Rio Negro*). Armadillos (*Cabasous unicinctus*; *Dasypus* spp.); Tapir (*Tapirus terrestris*); Paca (*Cuniculus paca*); Agoutis (*Dasyprocta fuliginosa* and *Dasyprocta leporina*); Deers (*Mazama americana* and *M. nemorivaga*); Acouchi (*Myoprocta acouchy*); and peccaries (*Pecari tajacu* and *Tayassu pecari*) were considered as a preferred target for hunters as a bushmeat source in our region. Although carnivorous and other predatory species (*Puma yagouaroundi*, *Leopardus pardalis*, *Panthera onca*, *Leopardus wiedii*, *Puma concolor* and *Eira barbara*) are also listed as the most hunted species in the study region (Campos, 2008; Pezzuti et al., 2004), they were treated as a separate group, as they are usually hunted for retaliation. *Proechimys* spp., *Philander opossum*, *Didelphis marsupialis*, *Sciurus igniventris*, *Metachirus nudicaudatus*, *Nasua nasua*, *Tamandua tetradactyla*, *Myrmecophaga tridactyla* and *Sciurus* spp. were considered as the least desirable targets because they were not preferred by hunter–gatherers as bushmeat (Campos, 2008; Pezzuti et al., 2004). Congener armadillos (*Dasypus* spp.), spiny rats (*Proechimys* spp.) and squirrels (*Sciurus* spp.) were each treated as a single taxon because of the difficulty in differentiating them on nocturnal (black and white) photos. We decided to include smaller species, such as squirrels, since a recent study in Peru showed that their detectability was higher with decreasing camera height (Whitworth et al., 2019). *Proechimys* spp. is a terrestrial species very well detected by camera traps with high relative abundance rates both in fluvial islands (Ferreira Neto et al., 2021) and *terra‐firme* forests (Gonçalves et al., 2022). The body mass of our sampled mammals was taken from cumulative research (Eisenberg & Redford, 1999; Emmons & Feer, 1997; Gonçalves et al., 2018; Reid, 1997), as were feeding guild preference and trophic level (Kissling et al., 2014). We classified species' body size as a continuous variable (Appendix S1).

### 2.4 Anthropogenic stressors

We obtained proxies for anthropogenic stressors: (i) poaching pressure, measured by the Euclidean shortest distance of each sampling station to the nearest human settlement (km) and (ii) the number of families per human settlement. Nowadays, although around 50 permanent communities exist around the study region, the settlements of Nova Esperança (58 families), Sobrado (87 families), Aracari (18 families), Madadá (1 family), Airão Velho (7 families) and Seringalzinho (12 families) were the nearest settlements to our sampling sites. Overall, there are a mean of four members in each family. This information was obtained under the author's request for the most recent census data from the Secretary of Amazonas State for the Environment (SEMA).

### 2.5 Data analysis

To explore the influence of anthropogenic predictors (distance from the nearest human settlement (km) (mean ± [SD] = 8.27 ± [3.92]; range = 4.03–17.7) and number of families per settlement (mean ± [SD] = 35.12 ± [27.61]; range = 1–87)), we performed single‐season occupancy and detection analyses following MacKenzie et al. (2002). As the total number of sampling days varied from 69 to 78 days, we combined detections into seven sampling occasions, each one spanning from 9 to 11 days per occasion, to build the detection history for each site. We coded whether the species was recorded (1) or not (0) by each camera trap on each sampling occasion. Our models consisted of two parameters: the occupancy probability (Ψ), defined as the probability of a site (in our case, each camera site) being occupied by species from different groups (in our case, the different groups of poaching preferences); and the detectability (*p*), defined as the probability of detecting different target species in a camera trap site during a specific time (or sampling occasion), given the site is occupied and that the detectability is less than 1 (Mackenzie et al., 2002).

We used the unmarked package (Fiske & Chandler, 2011) to fit single‐season occupancy models for each species group. We first used the function scale for standardising data for continuous variables. Subsequently, we evaluate the most parameterised model for overdispersion using the goodness‐of‐fit test developed for occupancy analyses based on 10,000 smoothed bootstraps (MacKenzie & Bailey, 2004) in the AICcmodavg package (Mazerolle, 2020). The models for the bushmeat and retaliation groups did not show overdispersion ($\hat{c}$ < 1 and *p* > .05). However, the most parameterised model for the non‐hunted group showed overdispersion ($\hat{c}$ = 3.81; *p* < .001). To correct for overdispersed count data for this last model, we used quasi‐AIC (QAICc) as a metric for model parsimony (Anderson et al., 1994). We built 18 models for each mammal group, including our a priori hypotheses. Specifically, we built either univariate (i.e. using either distance from settlements or number of families) or interaction (species body weight * distance to communities, species body weight * number of families and number of families * distance to settlements) models for psi, while modelling *p* as a function of either the number of days the cameras operated at each sampling occasion for each site or a univariate effect of species body weight. We also included the intercept‐only model structures [psi (.) *p* (.)], i.e., null models, for each model selection. We only considered models with Δ AICc ≤2 or QAICc ≤2, as likely to influence our parameters of interest. We used the most parsimonious models encompassing each variable of interest to extract beta values (and their respective SE's and 95% CI's) and final estimates (Appendix S2). Furthermore, we also used the ggplot2 (Wickham, 2016), jtools (Long, 2019), interactions (Long, 2022) and raster (Hijmans & Van Etten, 2012) packages to create graphs for the variables that affected our parameters of interest. All analyses were performed in the free R software (R Core Team, 2022).

## 3 RESULTS

### 3.1 Species richness and number of records

**FIGURE 2:**
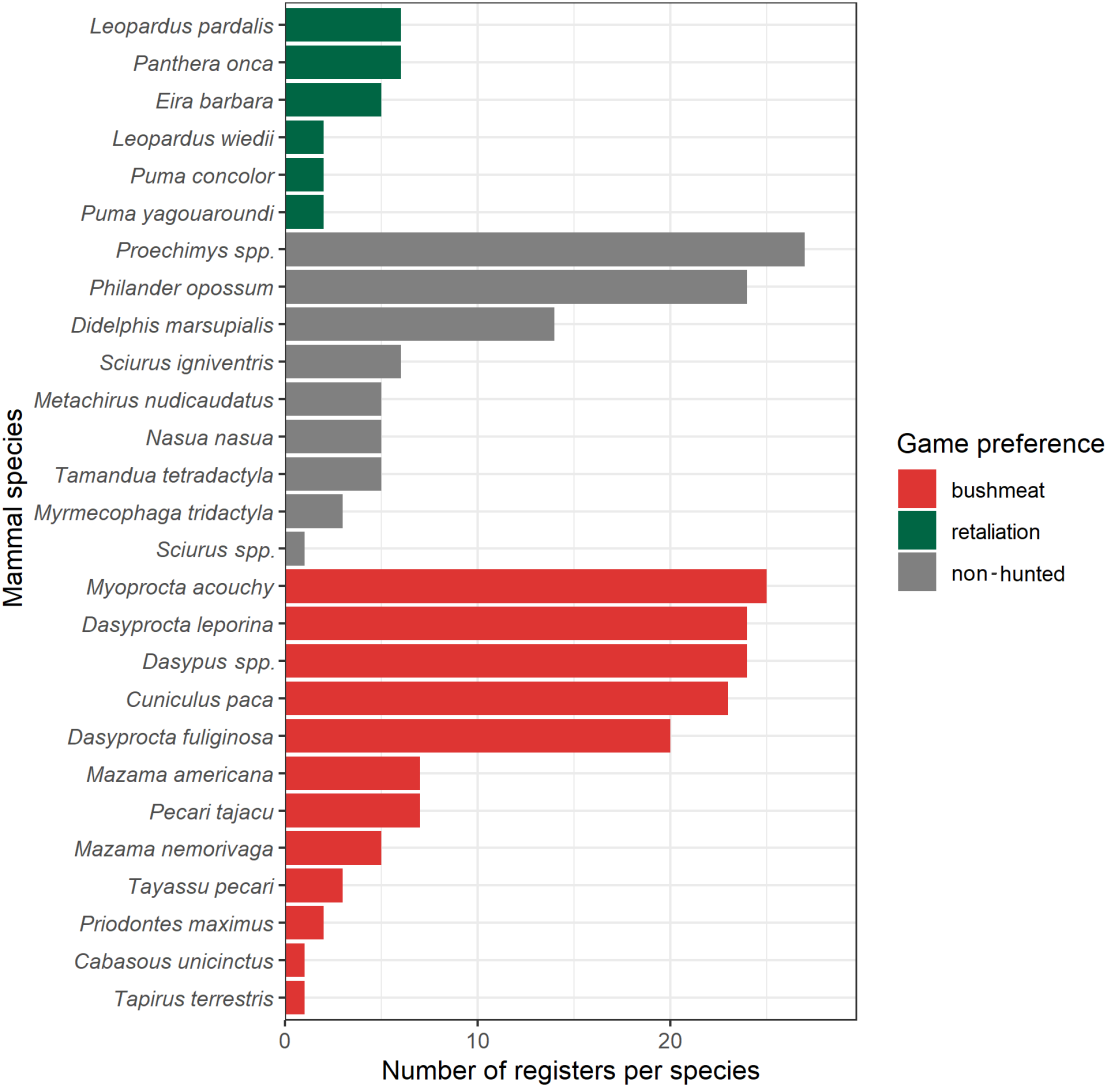
Number of records of 27 forest‐floor mammal species separated by colour according to game preference, with those most hunted for bushmeat (red), retaliation (green) and non‐hunted (grey).

We recorded 27 terrestrial mammal species through camera traps (Figure 2; Appendix S3). Three additional primate species that are predominantly arboreal (*Saimiri sciureus*, *Sapajus apella* and *Cebus albifrons*) and other small rodents (i.e. *Makalata* spp.) were registered, but since they were not efficiently detected by the camera trap method, they were not included in our analyses. Body weights ranged from less than 1 kg (*Metachirus nudicaudatus*, *Philander opossum*, *Proechimys* spp., *Sciurus igniventris* and *Sciurus* spp.) to more than 200 kg (*Tapirus terrestris*). Additionally, seven species from Rodentia, seven species from the Carnivora, four species from Artiodactyla, three species from Cingulata, three species from Didelphimorphia, two species from Pilosa and one species from Perissodactyla were classified.

### 3.2 Occupancy (Ψ) probabilities

Mammal occupancy showed different patterns according to hunters' preferences (Appendix S4). Anthropogenic stressors and species body weight influenced the occupancy probability of the bushmeat group (Table 1).

**TABLE 1:** Most parsimonious models (Δ ≤ 2; and their respective AICc weights) of different mammals according to game preferences (species hunted for bushmeat, species hunted for retaliation and non‐hunted species) in 47 *terra‐firme* sites in the Central Amazon.

Bushmeat				
Model	AICc	ΔAICc	AIC weights	Parameters
Ψ (families * distance), *p* (weight)	1793.44	0.00	0.45	6
Ψ (distance), *p* (weight)	1794.48	1.03	0.27	4
Ψ (weight * distance), *p* (weight)	1794.89	1.44	0.22	6

Retaliation				
Model	AICc	ΔAICc	AIC weights	Parameters
Ψ (.), *p* (.)	277.77	0.00	0.20	2
Ψ (.), *p* (days)	278.91	1.14	0.12	3
Ψ (families), *p* (.)	279.72	1.94	0.08	3
Ψ (distance), *p* (.)	279.76	1.99	0.08	3

Non‐hunted (c‐hat estimate = 3.81)				
Model	QAICc	ΔQAICc	QAICc weights	Parameters
Ψ (.), *p* (weight)	316.20	0	0.44	4
Ψ (families), *p* (weight)	317.95	1.76	0.18	5

*Note*: Occupancy probability (Ψ) was modelled as a function of the number of families at each settlement (families), the shortest distance between the camera site and human settlements (distance) and body mass (weight). The detection probability (*p*) was modelled as a function of the survey effort (number of days the cameras operated) and body mass (weight). The dot (.) signal means an intercept‐only model structure. * define the interaction between two predictors.

Distance to human settlements alone explained 27% (AICc weight of the univariate model = 0.27) of the effect on the occupancy probability of the bushmeat group. The interaction between this predictor and the number of families explained 45% (AICc weight of the interaction model = 0.45), which means that this interaction model is 0.45/0.27 ≈ 2 times more likely than the univariate model. Specifically, the occupancy probability of the bushmeat group was higher at sites away from human settlements.

**FIGURE 3:**
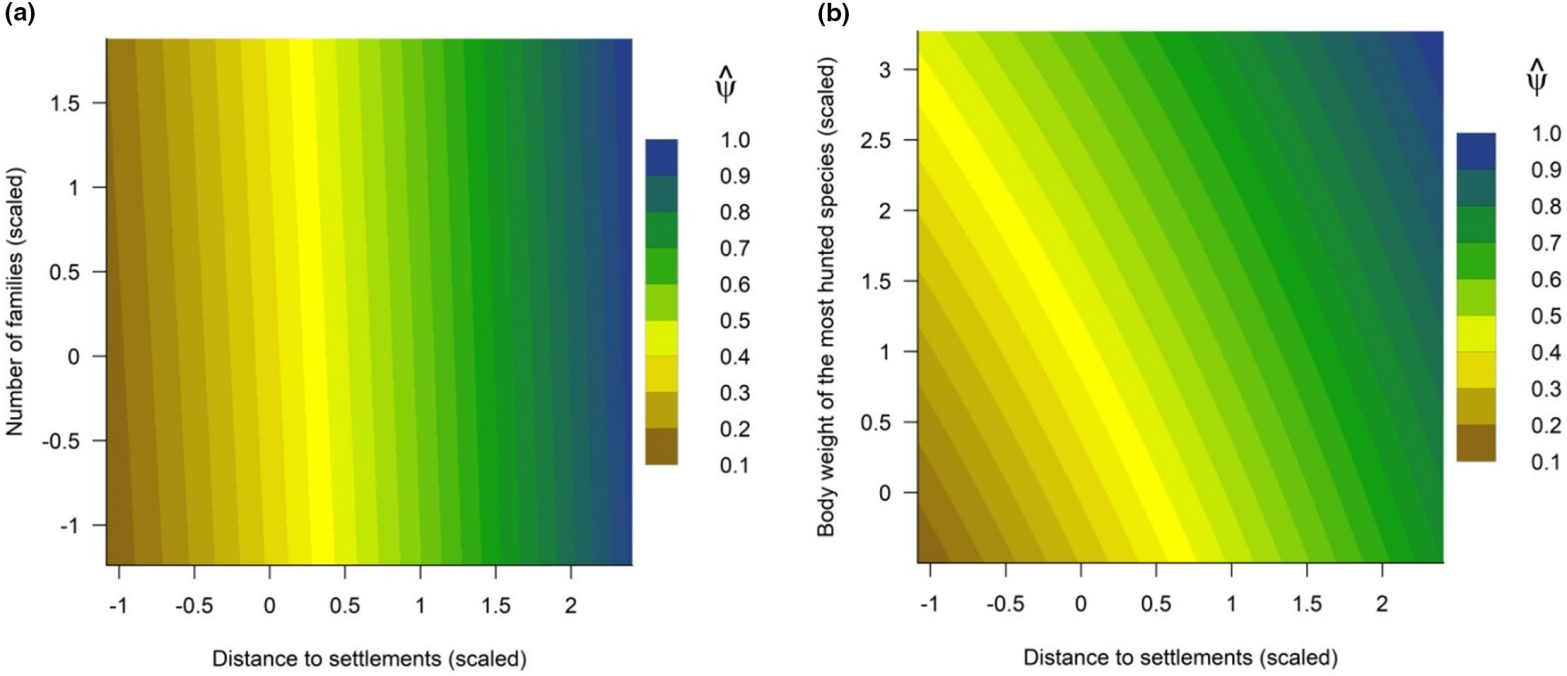
Occupancy probability of species hunted for bushmeat as a function of the interaction between distance to human settlements and number of families (a) and distance to settlements and species body weight (b) in *terra‐firme* forests of Central Amazonia. Estimates are from the most parsimonious models that included the predictor variables of interest.

Still, the occupancy probability of the bushmeat group at these sites was slightly higher when the number of families was larger (Figure 3a). However, the influence of the distance from human settlements on species occupancy probability was stronger than the influence of the number of families, as the occupancy probability was very high (>0.80) only at sites away from human settlements. Additionally, the occupancy probability of the group was influenced by the interaction between species body weight and distance from settlements, even though this interaction model was less probable (AICc weight of this interaction model = 0.22) than the other parsimonious models. Specifically, medium‐sized species (1–5 kg) increased their occupancy probabilities as the distance to settlements increased, and the same happened with large (5–15 kg) and very large (>15 kg) species. Still, large and very large species were more likely to occupy locations closer to human settlements than medium‐sized species. In other words, large and very large species were more evenly distributed across the gradient of anthropic pressures than small to medium‐sized species (Figure 3b).

For the species hunted due to retaliation, the number of families and distance to human settlements had a minimal influence on the occupancy probability of the group. The null model [psi (.)] was among the most parsimonious models. Specifically, the null model was 0.32 (i.e. the cumulative AICc weights for the two best‐ranked models that included the null model)/0.08 (i.e. the AICc weight of the univariate model that included the number of families and distance to the human settlements) = 4 times more likely than the model with the variables of interest. The mean occupancy probability of the group was 0.31 (IC‐95% = 0.09–0.68) for our studied area. The same pattern was observed for the group of the non‐hunted species, where the null model was among the most parsimonious models (ΔAICc < 2), which means that the predictor variables did not influence the occupancy probability of this group for our studied area. The mean occupancy probability of the group was 0.26 (IC‐95% = 0.21–0.31) for our studied area (Table 2).

**TABLE 2:** Mean occupancy probability and detection for our three groups (bushmeat, retaliation and non‐hunted).

Mean occupancy probability	Mean detection probability
*Bushmeat*	
0.49 (IC‐95% = 0.42–0.57)	0.49 (IC‐95% = 0.27–0.72)
*Retaliation*	
0.31 (IC‐95% = 0.09–0.68)	0.04 (IC‐95% = 0.01–0.11)
*Non‐hunted (c‐hat estimate = 3.81)*	
0.26 (IC‐95% = 0.21–0.31)	0.27 (IC‐95% = 0.21–0.35)

### 3.3 Detection (*p*) probabilities

**FIGURE 4:**
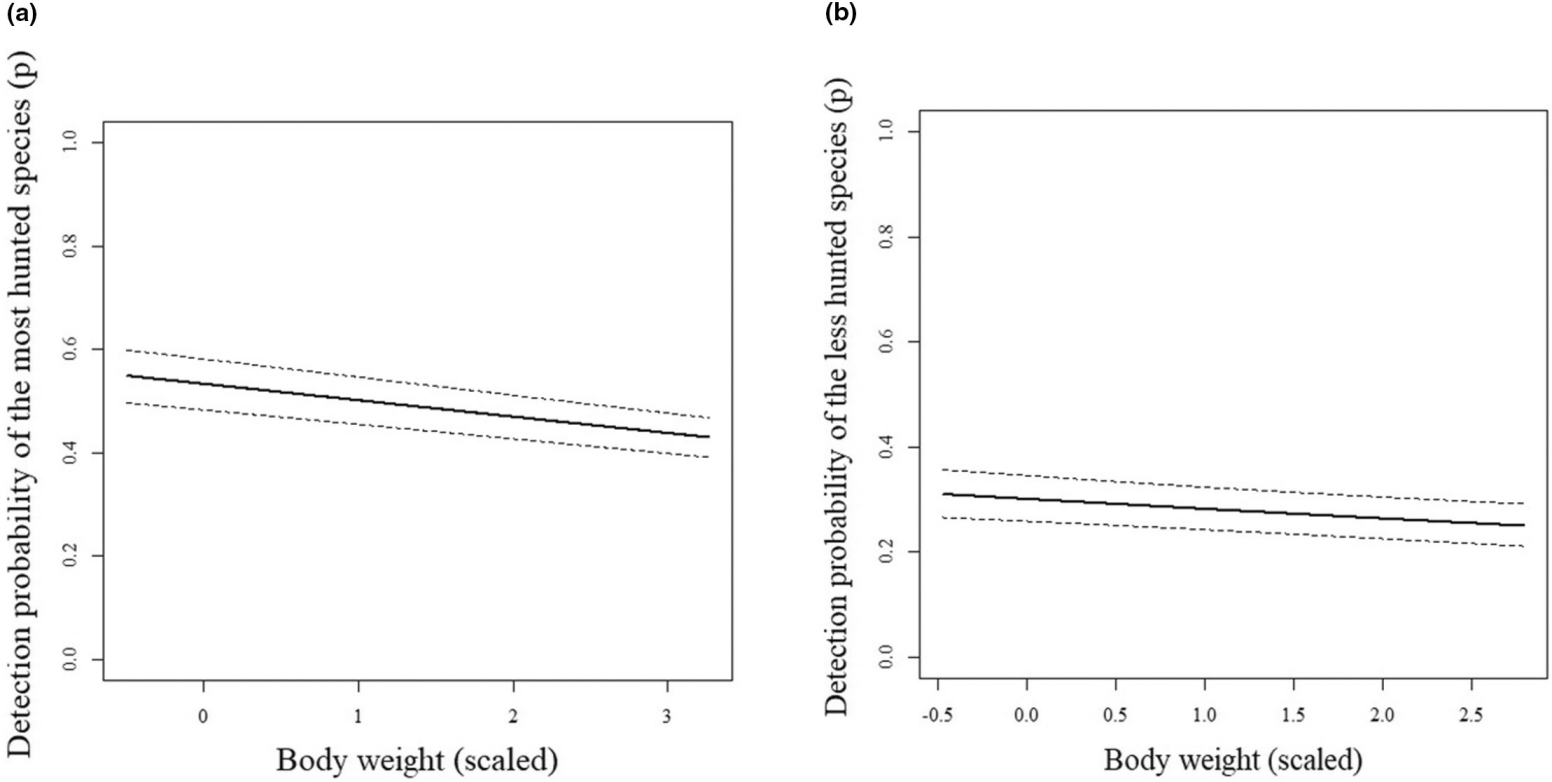
Detection probability (±95% CI) of the species hunted for bushmeat (a) and non‐hunted species (b) as a function of species body weight in *terra‐firme* forests of the Central Amazon. Estimates are from the models that included the variable of interest.

Detection probability decreased with body mass, with this effect being stronger for the bushmeat species than for the non‐hunted species (Figure 4a,b, respectively). For species hunted for retaliation, the null model was at least 0.36 (i.e. cumulative AICc weights of the models with the null structure)/0.12 (i.e. the AICc weight of the model with the survey effort) = 3 times more likely than a model with a predictor variable of interest. The mean detection probability of the group was 0.04 (IC‐95% = 0.01–0.11).

## 4 DISCUSSION

As far as we know, this study is the first attempt to underscore the synergetic influence of proxies of anthropogenic activities interacting with body mass on game preference in the oligotrophic ecosystems of the Central Amazon. As expected, mammals hunted for bushmeat were more commonly found far from settlements. We also expected that overhunting of the most depleted species could have been beneficial for the least hunted species, such as opossums, spiny rats, squirrels and anteaters. However, neither the occupancy of carnivorous species nor the less depleted species was affected by anthropogenic stressors. In addition, the detection probability of the most hunted species declined as body weight increased, suggesting that larger species from this group are rarer and, thus, less detected. The detection probability of the non‐hunted species also decreased. However, it was minimal, suggesting that even larger species from this group are more common than the bushmeat group, probably because they might be less hunted.

Several factors might explain the high occupancy probability of the bushmeat group at sites far from human settlements and a slight increase in the occupancy probability of the group at these sites when the number of families is higher. First, generally, hunters prefer to hunt target prey closer to where they live for several reasons, including easier access and lower costs (Parry et al., 2009). Also, locals rely on small farm areas to cultivate crops such as manioc and other fruiting trees (i.e. açai berry, peach palm and cupuaçu), drawing wildlife's attention (Campos, 2008). The arrangement of these fruiting trees and their management have been commonly used for decades by the human community as killing zones for poaching, even though some mammals could cause crop damage, as reported in the study region (Campos, 2008) and in the Western Brazilian Amazonia (Abrahams et al., 2018). Therefore, the mammal community of the bushmeat group might be less distributed at sites closer to these areas, with only a slight increase in occupancy, probably due to larger and more abundant crops and fruiting trees that still attract wildlife when the number of families is higher.

Contrary to what was predicted, we found a higher occupancy probability of larger than smaller species closer to settlements. One possible explanation is a change in bushmeat preference over time, which may be related to the depletion of some current target species close to human settlements. The most hunted species in 2008 were medium‐sized mammals, such as lowland pacas, and large‐sized mammals, such as peccaries (Campos, 2008). However, a recent interview in 2021 in the same study region (Amazonas, 2022) showed that the most hunted mammals were smaller‐sized species, such as agoutis, which may suggest a decrease in the abundance of smaller species and a slight recovery of larger species closer to human settlements. The occupancy probability of carnivores was not affected by distance to settlements or the number of families, contrary to what we expected. As carnivores have naturally low detection elsewhere concerning other groups (Foster & Harmsen, 2012), it can also affect the robustness of the results, particularly by inflating the occupancy probability estimates (MacKenzie et al., 2002). The carnivores could be the focus of long‐term surveys in future studies that will allow more robust inferences concerning the effects of anthropogenic stressors and retaliation hunting on their distribution in the study region. We also found that the occupancy probability of the non‐hunted species was not affected by distance to settlements and number of families, which means that they occur independently of these proxies of anthropogenic stressors. One possible explanation is that these species are avoided for hunting due to a cultural taboo or taste (Gutiérrez‐Granados & Dirzo, 2021; Melo et al., 2015).

Some species are under higher pressure since they are also the most hunted locally and regionally in different parts of the Amazon. In the study region, agoutis, deer, peccaries, armadillos and tapirs are considered ‘good for hunting’ (Campos, 2008; Pezzuti et al., 2004). At the same time, other species such as the giant and lesser anteater, coati, some small mammals and felids are avoided as bushmeat by some locals of the study area because of the taste or a cultural taboo (Campos, 2008; Pezzuti et al., 2004).

One of the factors that could mitigate the impact of anthropogenic pressures is forest productivity (Ferreira Neto et al., 2021). In the study area, sites closer to settlements but with greater productivity had a higher number of records and species of mammals compared with lower‐productivity sites (Ferreira Neto et al., 2021). It is suggested that more fertile soils experience greater ecosystem turnover, which reduces the effect of human disturbance factors and increases the chance of individual and species survival via higher productivity (de Souza Ferreira Neto et al., 2021, 2022). Even smaller variations in oligotrophic soils explain floristic composition in the Central Amazon (Campos, 2017). Considering the bottom‐up productivity force, we thus suggest that these productivity measures should be investigated in further studies for *terra‐firme* forests, since higher soil fertility may weaken the anthropic disturbance of mammals through higher investment in plant reproduction (Chave et al., 2010) and by increasing foliage nutrient content, making them more palatable for herbivores (Coley et al., 1985; Vitousek, 1984). Hence, increasing productivity with resource availability is more likely to increase the biomass and number of individuals per species, decreasing the pervasive effect of anthropic disturbances (Peres, 2008), since higher productivity could give more resilience to wild meat harvest (Ferreira Neto et al., 2021).

We provided consistent camera trap registers, recording at least 27 forest‐floor mammal species in our sampled area. Among these, 21 species were considered least concern, according to the IUCN. Still, some of our hunted species are on the red list of the IUCN in different threat categories. The red brocket deer is classified as the data deficient (Duarte & Vogliotti, 2016); the jaguar (Quigley et al., 2017); and the margay (Oliveira et al., 2015) as near threatened; while the giant anteater (Miranda et al., 2014), giant armadillo (Anacleto et al., 2014), tapir (Varela et al., 2019) and white‐lipped peccary are classified as Vulnerable (Keuroghlian et al., 2013). Therefore, they might be vulnerable to extinction, facing rapid and continuous population decline. Other species were not registered in this study in *terra‐firme* forests but were registered in neighbouring fluvial islands, such as capybaras (*Hydrochoerus hydrochaeris*) and Brazilian porcupine (*Coendou prehensilis*) (Ferreira Neto et al., 2021). Indeed, according to the current study, *terra‐firme* forests harbour more than twice as many species from similar sampling efforts as fluvial islands (Ferreira Neto et al., 2021; Neto et al., 2022).

Game species can be an essential source of income for rural Amazonians, although it is an illegal trade. In the Colombia, Peru and Brazil border regions, the average price for chicken is one‐quarter of bushmeat and wild meat is sold at similar prices to beef meat (Van Vliet et al., 2014). Since bushmeat is economically affordable for locals, one possible alternative to mitigate the impacts of poaching in the ecosystem is the domestication of some species, such as paca, which rural Amazonians greatly appreciate. Still, it is also widely distributed in the Neotropic region (Van Vliet et al., 2014) and less vulnerable to extinction than other mammals, such as larger primates and lowland tapirs (Bodmer et al., 2020). Another alternative for a more sustainable harvest that could also decrease the impact of poaching on terrestrial mammals comes from a study with cracid birds, which has suggested that the effect of poaching on the latter might decrease if there is a surrounding unhunted population and if hunting is occasional (Begazo & Bodmer, 1998). On the contrary, the illegal and indiscriminate commercialisation of bushmeat represents a greater impact on wildlife, which not only will severely affect the occupancy probability of mammals but also poses a threat to food security for rural Amazonians since game meat for subsistence is a vital source of protein for isolated rural communities (El Bizri et al., 2020).

Bushmeat is an essential part of rural Amazonian culture and an important source of protein and income. Generally, bushmeat for subsistence (i.e. wildlife used for human consumption) is more common in this region (Campos, 2008). Still, the illegal trade for mammals has also been reported, especially salt bushmeat of tapirs and peccaries, sold in the settlements surrounding the protected areas and the neighbouring city of Novo Airão (Pezzuti et al., 2004). In the Equatorial Guinea, bushmeats are also sold in luxury restaurants for the wealthy class (Fa et al., 2009). In our study region, the hunters use firearms (i.e. usually shotguns) combined with various techniques, mainly killing in ambushes and sometimes by using dogs, depending on the targeted species (Pezzuti et al., 2004). Buying and selling wildlife violates legislation because hunting in some protected areas, such as the Anavilhanas and Jaú National Park, is not allowed. Hence, the Fauna Protection Law (No. 5197 of January 1967) and the Federal Law of Environmental Crimes (No. 9605 of February 12, 1998) conceptualise hunting as a non‐bailable crime, except for cases of extreme need for food sources or in Indigenous territories.

Bushmeat for subsistence is cultural and necessary as a source for protein supplementation. Accounting for the basic need for protein and the health of locals, future studies could take a step further and focus on improving our understanding of species game preference, poaching types, techniques and strategies, cultural taboos and species avoided. In addition, how poaching is performed, the main reason for clandestine hunting and the relationship of all these to the nutrition profile of locals (i.e. if it is for bushmeat, retaliation or use of animal parts). The knowledge gained would help to detect the influence of poaching and safeguard forest‐floor species in oligotrophic ecosystems (Melo et al., 2015).

## 5 ENVIRONMENTAL POLICY IMPLICATION

Our study serves as an important reminder about the sustainability of the possible current harvest in the study area since the anthropogenic proxies' stressors had a strong negative effect on the occupancy probability of bushmeat species for all mammal sizes but not for the other groups (non‐hunted and carnivores). The detection probability for the non‐hunted and especially for the bushmeat group species declined slightly, suggesting that larger species, particularly those from the bushmeat group, are rarer in the system, likely because they combine lower growth rates and are more depleted. Sites closer to human settlements have a low occupancy probability for the most hunted species of all mammal sizes. However, even though these sites may still provide bushmeat for locals, it is more likely that in the long term, locals will need to travel longer distances to find harvest meat, which means that "garden hunting" will need to be replaced for greater investment in economic resources and time. In other words, the local strategy of attracting wildlife closer to settlements with fruiting trees may not be enough since the occupancy probability is lower near settlements. Overhunting can lead in the long term to the erosion of animal biomass and the empty‐forest effect, which means that the forest may seem intact, but several ecological functions and trophic webs could have been already disrupted (Benítez‐López et al., 2019; Hughes et al., 2022). Thus, the disappearance of tropical rainforest architects who act as ecological engineers may trigger several adverse cascading effects on the ecosystem (Dirzo et al., 2014; Lacher et al., 2019; Lavery et al., 2020; Pires et al., 2018; Villar et al., 2021).

Last, insights from our study further assist management decisions to guarantee species conservation and sustainable bushmeat for locals. For communities within and surrounding protected areas, we suggest promoting more sustainable alternatives for generating income such as ecotourism, minimising the impact of poaching on the conservation units and using conciliatory approaches that align environmental protection with local community needs through environmental education and intense surveillance activities with the involvement of locals by paying them. In addition, we urge governments to guarantee better social capital and living conditions for rural Amazonians to decrease poverty, which is expected to decrease the poaching pressure through a reduced wild meat trade. We strongly suggest future studies differentiate the magnitudes of direct poaching effects across different groups of hunted and non‐hunted species, such as species hunted for bushmeat and hunted for retaliation. In this context, our model approach might be an excellent tool to monitor temporal variation in mammal distributions and densities according to poaching proxies. Our results showed that measures of anthropogenic effects are robust and could be considered in conservation units, in regions that have been little studied and with fewer resources. In sum, our measures of anthropogenic factors influenced the probability of occupancy and detection of mammals, especially those most hunted for bushmeat.

## AUTHOR CONTRIBUTIONS

**Gilson de Souza Ferreira Neto:** Conceptualization (lead); data curation (lead); formal analysis (lead); funding acquisition (lead); investigation (lead); methodology (lead); project administration (lead); resources (lead); software (lead); supervision (lead); validation (lead); visualization (lead); writing – original draft (lead); writing – review and editing (lead). **Fabricio Beggiato Baccaro:** Conceptualization (supporting); data curation (supporting); formal analysis (supporting); funding acquisition (supporting); investigation (supporting); methodology (supporting); project administration (supporting); resources (supporting); software (supporting); supervision (supporting); validation (supporting); visualization (supporting); writing – original draft (supporting); writing – review and editing (supporting). **Matthew J. Phillips:** Writing – review and editing (supporting). **Rodrigo Lima Massara:** Conceptualization (supporting); data curation (supporting); formal analysis (supporting); funding acquisition (supporting); investigation (supporting); methodology (supporting); project administration (supporting); resources (supporting); software (supporting); supervision (supporting); validation (supporting); visualization (supporting); writing – original draft (supporting); writing – review and editing (supporting).

## FUNDING INFORMATION

CAPES/Doutorado‐Sanduíche no exterior, Grant/Award Number: (PDSE; # 88881.690164/2022‐01); Rufford Foundation (grant number 30394‐2); Idea Wild for instruments support.

## CONFLICT OF INTEREST STATEMENT

The authors declare no conflict of interest.

## Supporting information


Appendix S1–S4.

## Data Availability

All data supporting this study's findings are available in this article's supporting information.
