# Supplementary material for: The distribution of bushmeat mammals in unflooded forests of the Central Amazon is influenced by poaching proxies
Source: Ecol Evol. 2023 Dec 3;13(12):e10783. doi: 10.1002/ece3.10783 (PMC10694382; doi:10.1002/ece3.10783)
Supplement: Supplementary file 1 — Appendix S1–S4. [file ECE3-13-e10783-s001.docx]

**SUPPORTING INFORMATION**

Appendix S1. Characteristics of the species accordingly to body mass, feeding guild, Order, game preference, and IUCN conservation status.

| **Species** | **Common name** | **Body mass (kg)** | **Feeding guild** | **Order** | **Game preference** | **IUCN Red List status** |
| --- | --- | --- | --- | --- | --- | --- |
| *Cabasous unicinctus* | southern naked-tailed armadillo | 3 | Insectivorous | Pilosa | bushmeat | Least Concern |
| *Cuniculus paca* | lowland paca | 9 | Frugivorous-Herbivorous | Rodentia | bushmeat | Least Concern |
| *Dasyprocta fuliginosa* | black agouti | 3.5 | Frugivorous-Herbivorous | Rodentia | bushmeat | Least Concern |
| *Dasyprocta leporina* | red-rumped agouti | 3 | Frugivorous-Herbivorous | Rodentia | bushmeat | Least Concern |
| *Dasypus spp.* | long-nosed armadillo | 2 | Insectivorous | Cingulata | bushmeat | Least Concern |
| *Didelphis marsupialis* | common opossum | 1. 5 | Omnivorous | Didelphimorphia | less hunted | Least Concern |
| *Eira barbara* | Tayra | 4.8 | Omnivorous | Carnivora | retaliation | Least Concern |
| *Puma yagouaroundi* | jaguarundi | 6.9 | Carnivorous | Carnivora | retaliation | Least Concern |
| *Leopardus pardalis* | ocelot | 10.3 | Carnivorous | Carnivora | retaliation | Least Concern |
| *Leopardus wiedii* | margay | 3.6 | Carnivorous | Carnivora | retaliation | Near Threatened |
| *Mazama americana* | Red Brocket Deer | 36 | Frugivorous-Herbivorous | Artiodactyla | bushmeat | Data Deficient |
| *Mazama nemorivaga* | Amazonian brown brocket | 28 | Frugivorous-Herbivorous | Artiodactyla | bushmeat | Least Concern |
| *Metachirus nudicaudatus* | brown four-eyed opossum | 0.382 | Omnivorous | Didelphimorphia | less hunted | Least Concern |
| *Myoprocta acouchy* | red acouchi | 1 | Frugivorous-Herbivorous | Rodentia | bushmeat | Least Concern |
| *Myrmecophaga tridactyla* | giant anteater | 37 | Insectivorous | Pilosa | less hunted | Vulnerable |
| *Nasua nasua* | South American coati | 3.9 | Omnivorous | Carnivora | less hunted | Least Concern |
| *Panthera onca* | jaguar | 76 | Carnivorous | Carnivora | retaliation | Near Threatened |
| *Pecari tajacu* | collared peccary | 21.7 | Frugivorous-Herbivorous | Artiodactyla | bushmeat | Least Concern |
| *Philander opossum* | gray four-eyed opossum | 0.437 | Omnivorous | Didelphimorphia | less hunted | Least Concern |
| *Priodontes maximus* | giant armadillo | 26 | Insectivorous | Cingulata | bushmeat | Vulnerable |
| *Proechimys spp.* | spiny rat | 0.5 | Omnivorous | Rodentia | less hunted | Least Concern |
| *Puma concolor* | puma | 63 | Carnivorous | Carnivora | retaliation | Least Concern |
| *Sciurus igniventris* | Amazon red squirrel | 0.615 | Frugivorous-Herbivorous | Rodentia | less hunted | Least Concern |
| *Sciurus spp.* | Brazilian squirrel | 0.39 | Frugivorous-Herbivorous | Rodentia | less hunted | Least Concern |
| *Tamandua tetradactyla* | lesser anteater | 4.9 | Insectivorous | Pilosa | less hunted | Least Concern |
| *Tapirus terrestris* | South American Tapir | 225 | Frugivorous-Herbivorous | Perissodactyla | bushmeat | Vulnerable |
| *Tayassu pecari* | white-lipped peccary | 33 | Frugivorous-Herbivorous | Artiodactyla | bushmeat | Vulnerable |

Appendix S2. The beta and their respective SE's e 95% CI's estimates were extracted from the best parsimonious models that included the variables of interest of different mammals accordingly with game preference (hunted for bushmeat, hunted for retaliation and non-hunted) in 47 terra firme sites in Central Amazon.

|  | ***B* parameters** | | | |
| --- | --- | --- | --- | --- |
| **Covariate** | **Estimate** | **SE's** | **Lower**  **95% CI** | **Upper 95% CI** |
| **More hunted species occupancy (Ψ)** |  |  |  |  |
| Distance from communities | 0.266 | 0.117 | 0.036 | 0.496 |
| Number of families | 0.039 | 0.114 | -0.183 | 0.262 |
| Distance from communities:number of families | -0.258 | 0.138 | -0.528 | 0.012 |
| Distance from communities:weight | -1.778 | 0.939 | -3.619 | 0.062 |
| Number of families:weight | 1.927 | 0.825 | 0.31 | 3.542 |
| **More hunted species detection (p)** |  |  |  |  |
| Days of camera operation | -0.046 | 0.07 | -0.184 | 0.091 |
| Weight | -7.460 | 0.706 | -8.848 | -6.078 |
| **Carnivorous occupancy (Ψ)** |  |  |  |  |
| Distance from communities | 0.017 | 0.074 | -0.129 | 0.163 |
| Number of families | -0.003 | 0.01 | -0.023 | 0.017 |
| Distance from communities:number of families | 0.003 | 0.002 | -0.001 | 0.008 |
| Distance from communities:weight | -0.002 | 0.004 | -0.010 | 0.006 |
| Number of families:weight | -0.0008 | 0.0002 | -0.001 | -0.0003 |
| **Carnivorous detection (p)** |  |  |  |  |
| Days of camera operation | -0.375 | 0.395 | -1.147 | 0.399 |
| Weight | -0.0004 | 0.007 | -0.014 | 0.013 |
| **Non-hunted species occupancy (Ψ)** |  |  |  |  |
| Distance from communities | -0.0002 | 0.0322 | -0.063 | 0.062 |
| Number of families | -0.0049 | 0.00463 | -0.013 | 0.004 |
| Distance from communities:number of families | -0.003 | 0.107 | -0.271 | 0.151 |
| Distance from communities:weight | -0.013 | 0.0132 | -0.039 | 0.012 |
| Number of families:weight | 0.0004 | 0.0015 | -0.002 | 0.003 |
| **Non-hunted species detection (p)** |  |  |  |  |
| Days of camera operation | -0.120 | 0.172 | -0.457 | 0.217 |
| Weight | -0.0921 | 0.0242 | -0.139 | -0.044 |

Appendix S3. Camera trap registers recorded in *terra-firme* forests in Central Amazon of A) *Leopardus pardalis*; B) *Mazama americana*; C) *Eira barbara*; D) *Myrmecophaga tridactyla*; E) *Herpailurus yagouaroundi*; and F) *Panthera onca*.


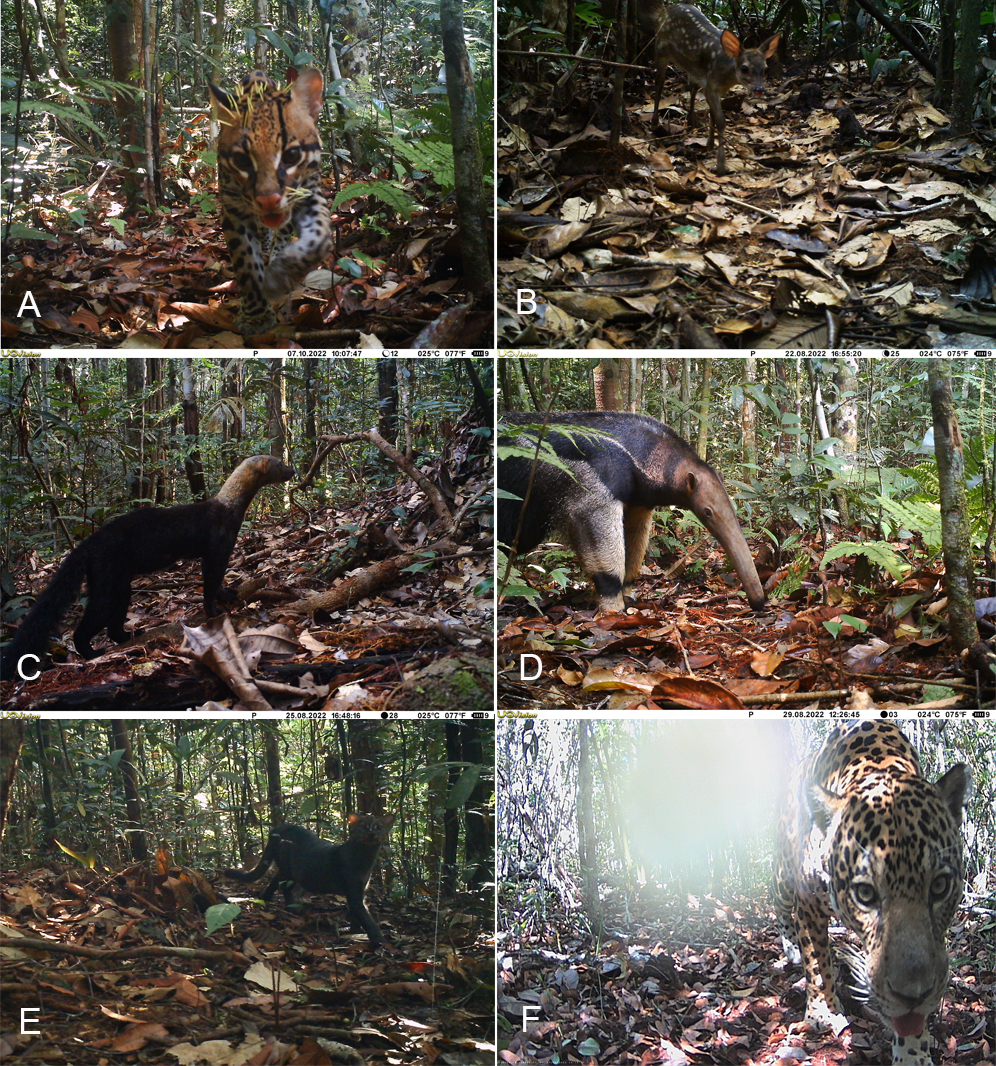


Appendix S4. Table of model results used to model the probabilities of occupancy (Ψ) and detection (p) of different mammals accordingly with game preference (hunted for bushmeat, hunted for retaliation, and non-hunted) in 47 *terra-firme* sites in Central Amazon. Occupancy probability was modelled as a function of the number of families at each settlement (families), the shortest distance between the camera site and human settlements (distance), and body mass (weight). Detection probability was modelled as a function of the survey effort (days) and body mass (weight). The dot (.) signal means an intercept-only model structure.

| **Bushmeat** | | | | |
| --- | --- | --- | --- | --- |
| **Model** | **AICc** | **Δ AICc** | **AIC weights** | **Parameters** |
| Ψ (families * distance) p (weight) | 1793.44 | 0.00 | 0.45 | 6 |
| Ψ (distance) p (weight) | 1794.48 | 1.03 | 0.27 | 4 |
| Ψ (weight * distance) p (weight) | 1794.89 | 1.44 | 0.22 | 6 |
| Ψ (.) p(weight) | 1797.80 | 4.35 | 0.05 | 3 |
| Ψ (families) p(weight) | 1799.71 | 6.26 | 0.02 | 4 |
| Ψ (weight * families) p(weight) | 1809.24 | 15.79 | 0.00 | 6 |
| Ψ (weight * distance) p(.) | 1868.42 | 74.98 | 0.01 | 5 |
| Ψ (weight * distance) p(days) | 1869.97 | 76.53 | 0.02 | 6 |
| Ψ (weight * families) p(.) | 1876.95 | 83.51 | 0.03 | 5 |
| Ψ (weight * families) p(days) | 1905.94 | 112.50 | 0.04 | 6 |
| Ψ (distance) p(.) | 1929.45 | 136.01 | 0.05 | 3 |
| Ψ (distance) p(days) | 1931.02 | 137.58 | 0.06 | 4 |
| Ψ (.) p(.) | 1931.20 | 137.76 | 0.07 | 2 |
| Ψ (.) p(days) | 1932.82 | 139.38 | 0.08 | 3 |
| Ψ (families) p(.) | 1932.99 | 139.55 | 0.09 | 3 |
| Ψ (families) p(days) | 1934.56 | 141.12 | 0.10 | 4 |
| Ψ (families * distance) p(.) | 1936.13 | 142.69 | 0.11 | 5 |
| Ψ (families * distance) p(days) | 1937.20 | 143.75 | 0.12 | 6 |
| **Retaliation** | | | | |
| **Model** | **AICc** | **Δ AICc** | **AIC weights** | **Parameters** |
| Ψ (.) p(.) | 277.77 | 0.00 | 0.20 | 2 |
| Ψ (.) p(days) | 278.91 | 1.14 | 0.12 | 3 |
| Ψ (families) p(.) | 279.72 | 1.94 | 0.08 | 3 |
| Ψ (distance) p(.) | 279.76 | 1.99 | 0.08 | 3 |
| Ψ (weight * distance) p(.) | 279.81 | 2.04 | 0.07 | 5 |
| Ψ (distance) p(days) | 280.51 | 2.74 | 0.05 | 4 |
| Ψ (families) p(days) | 280.80 | 3.03 | 0.04 | 4 |
| Ψ (families * distance) p(.) | 280.88 | 3.11 | 0.04 | 5 |
| Ψ (weight * distance) p(days) | 281.77 | 4.00 | 0.03 | 6 |
| Ψ (families * distance) p(days) | 281.82 | 4.05 | 0.03 | 6 |
| Ψ (weight * families) p(days) | 282.86 | 5.09 | 0.017 | 6 |
| Ψ (distance) p(weight) | 283.06 | 5.29 | 0.01 | 4 |
| Ψ (families) p(weight) | 283.62 | 5.84 | 0.01 | 4 |
| Ψ (.) p(weight) | 284.59 | 6.82 | 0.01 | 3 |
| Ψ (weight * distance) p(weight) | 284.80 | 7.03 | 0.01 | 6 |
| Ψ (weight * families) p(weight) | 285.69 | 7.92 | 0.00E+00 | 6 |
| Ψ (distance * families) p(weight) | 310.85 | 33.08 | 0.00E+00 | 6 |
| Ψ (weight * families) p(.) | 441.45 | 163.68 | 0.00E+00 | 5 |
| **Non-hunted (c-hat estimate = 3.81)** | | | | |
| **Model** | **QAICc** | **Δ QAICc** | **QAICc weights** | **Parameters** |
| Ψ (.) p(weight) | 316.20 | 0 | 0.44 | 4 |
| Ψ (families) p(weight) | 317.95 | 1.76 | 0.18 | 5 |
| Ψ (distance) p(weight) | 318.25 | 2.05 | 0.16 | 5 |
| Ψ(.) p(.) | 320.80 | 4.61 | 0.04 | 3 |
| Ψ (distance * weight) p(weight) | 321.94 | 5.75 | 0.03 | 7 |
| Ψ (weight * families) p(weight) | 322.05 | 5.85 | 0.02 | 7 |
| Ψ (distance * families) p(weight) | 322.40 | 6.20 | 0.02 | 7 |
| Ψ (families) p(.) | 322.56 | 6.36 | 0.02 | 4 |
| Ψ(.) p(days) | 322.71 | 6.51 | 0.02 | 4 |
| Ψ (distance) p(.) | 322.84 | 6.64 | 0.02 | 4 |
| Ψ (weight * distance) p(.) | 323.20 | 7.01 | 0.01 | 6 |
| Ψ (weight * families) p(.) | 323.47 | 7.27 | 0.01 | 6 |
| Ψ (families) p(days) | 324.53 | 8.33 | 0.01 | 5 |
| Ψ (distance) p(days) | 324.76 | 8.56 | 0.01 | 5 |
| Ψ (weight * distance) p(days) | 325.14 | 8.95 | 0.01 | 7 |
| Ψ (families * weight) p(days) | 325.48 | 9.29 | 0.00 | 7 |
| Ψ (families * distance) p(.) | 326.43 | 10.24 | 0.00 | 6 |
| Ψ (families * distance) p(days) | 329.06 | 12.86 | 0.00 | 7 |
